# Treatment Outcome and Prognostic Molecular Markers of Supratentorial Primitive Neuroectodermal Tumors

**DOI:** 10.1371/journal.pone.0153443

**Published:** 2016-04-13

**Authors:** Seo Hee Choi, Se Hoon Kim, Kyu-Won Shim, Jung Woo Han, Junjeong Choi, Dong-Seok Kim, Chuhl Joo Lyu, Jun Won Kim, Chang-Ok Suh, Jaeho Cho

**Affiliations:** 1 Departments of Radiation Oncology, Yonsei University College of Medicine, Seoul, Korea; 2 Departments of Pathology, Yonsei University College of Medicine, Seoul, Korea; 3 Departments of Neurosurgery, Yonsei University College of Medicine, Seoul, Korea; 4 Departments of Pediatrics, Yonsei University College of Medicine, Seoul, Korea; 5 Department of Pharmacy, College of Pharmacy, Yonsei University College of Medicine, Seoul, Korea; 6 Department of Radiation Oncology, Gangnam Severance Hospital, Yonsei University College of Medicine, Seoul, Korea; University Hospital of Navarra, SPAIN

## Abstract

**Background:**

To identify prognostic factors and define the optimal management of patients with supratentorial primitive neuroectodermal tumors (sPNETs), we investigated treatment outcomes and explored the prognostic value of specific molecular markers.

**Methods:**

A total of 47 consecutive patients with pathologically confirmed sPNETs between May 1985 and June 2012 were included. Immunohistochemical analysis of LIN28, OLIG2, and Rad51 expression was performed and correlated with clinical outcome.

**Results:**

With a median follow-up of 70 months, 5-year overall survival (OS) and progression-free survival (PFS) was 55.5% and 40%, respectively, for all patients. Age, surgical extent, and radiotherapy were significant prognostic factors for OS and PFS. Patients who received initially planned multimodal treatment without interruption (i.e., radiotherapy and surgery (≥subtotal resection), with or without chemotherapy) showed significantly higher 5-year OS (71.2%) and PFS (63.1%). In 29 patients with available tumor specimens, tumors with high expression of either LIN28 or OLIG2 or elevated level of Rad51 were significantly associated with poorer prognosis.

**Conclusions:**

We found that multimodal treatment improved outcomes for sPNET patients, especially when radiotherapy and ≥subtotal resection were part of the treatment regimen. Furthermore, we confirmed the prognostic significance of LIN28 and OLIG2 and revealed the potential role of Rad51 in sPNETs.

## Introduction

Primitive neuroectodermal tumors (PNETs) are the most frequent type of malignant pediatric brain tumors, which primarily consist of undifferentiated round neuroepithelial cells. PNETs include central nervous system (CNS) PNETs (also called supratentorial PNETs or sPNETs). Although sPNETs are histologically indistinguishable from medulloblastoma, multiple studies have identified different molecular characteristics between the two types of tumors, suggesting differences in their clinical prognosis [1, 2].

sPNETs are associated with poor prognosis, with a 5-year event-free survival rate of 30–60% depending on treatment modality. Certain factors, including young age, tumor dissemination, no radiotherapy, and no grossly total resection, are related to poorer prognosis, emphasizing the importance of multimodal treatment or tailored therapeutic strategies for each patient [3–5]. The standard treatment for sPNETs has typically been intensive, consisting of major surgical resection when feasible, followed by radiotherapy and chemotherapy [6–10].

Recently, molecular profiling is important for prognosis and selection of appropriate modality. However, the molecular composition of most sPNETs is unknown, and the prognostic impact of various pathologic and genetic factors has not been thoroughly investigated. Three molecular subgroups of sPNETs have been found to exhibit differential expression of cell-lineage markers LIN28 and OLIG2, and to be related to distinct demographic features [11, 12]. We tried to find other prognostic markers in sPNET. There are other molecular markers affecting resistance against radiation therapy and local failure, and Rad51 is one of these key proteins. Expression of Rad51, which plays a central role in the repair of DNA double-strand breaks, has been recently examined in many types of tumors [13] but not sPNETs. It is generally suggested that Rad51 overexpression increases cellular resistance to radiation and some chemotherapeutic drugs [14]. Rad51 overexpression is associated with poor prognosis for many cancers [15–18], with only two studies showing the opposite relationship [19, 20]. Thus, we suggested that this molecular marker could not only be used to predict prognosis but may also serve as potential therapeutic targets for sPNETs.

At our institution, patients with sPNETs have received multimodal treatment including surgery, radiotherapy, and chemotherapy depending on each patient’s risk factors and disease status. To identify prognostic factors and define optimal management of sPNET patients, we first investigated treatment outcomes at our institution. Second, we analyzed levels of LIN28, OLIG2, and Rad51 expression and correlated these expression levels with patient survival.

## Materials and Methods

### Patient characteristics

Fifty-four consecutive patients with pathologically confirmed sPNETs who were treated between May 1985 and June 2012 were retrospectively reviewed. Seven patients were excluded because of insufficient treatment information, follow-up loss, or uncertain pathology. Therefore, a total of 47 patients were included in the analysis. All patients were pathologically classified as sPNETs according to the revised WHO classification 2007. Patient characteristics are summarized in Table 1. The median follow-up duration was 70 months (range, 7 months-28 years), and the average follow-up duration was 59 months. Patients with a follow-up duration shorter than 1 year had all died, even at 7 months after diagnosis. This study was approved by Institutional review board (IRB) of Yonsei University Health System. The patient records/information was anonymized and de-identified prior to analysis, and informed consent was not obtained from each participants.

**Table 1:** Patient characteristics.

Variables	No.	%
**Sex**		
** M**	19	40
** F**	28	60
**Age**		
** Median (months)**	140	
** Range (months)**	1–828	
** < 3 years**	7	15
** ≥ 3 years**	40	85
**Initial KPS**		
** < 80**	22	47
** ≥ 80**	25	53
**Tumor location**		
** Pineal gland**	14	30
** Non-pineal gland**	33	70
**Multiplicity**		
** Yes**	3	6
** No**	44	94
**Metastasis**		
** Yes**	12	26
** No**	35	74

Abbreviations: KPS, Karnofsky performance status

### Treatment

Various treatment characteristics were analyzed, including treatment modality, extent of resection, use of radiotherapy, and use of chemotherapy. Surgical extent was classified as grossly total resection (GTR), subtotal resection (STR), or partial resection (PR). Subtotal and partial resection was defined as resection of a gross tumor by more or less than 90%, respectively. Surgical extent was determined according to the surgeon’s operation record and MRI scans conducted within 48 hours after surgery.

**Fig 1:**
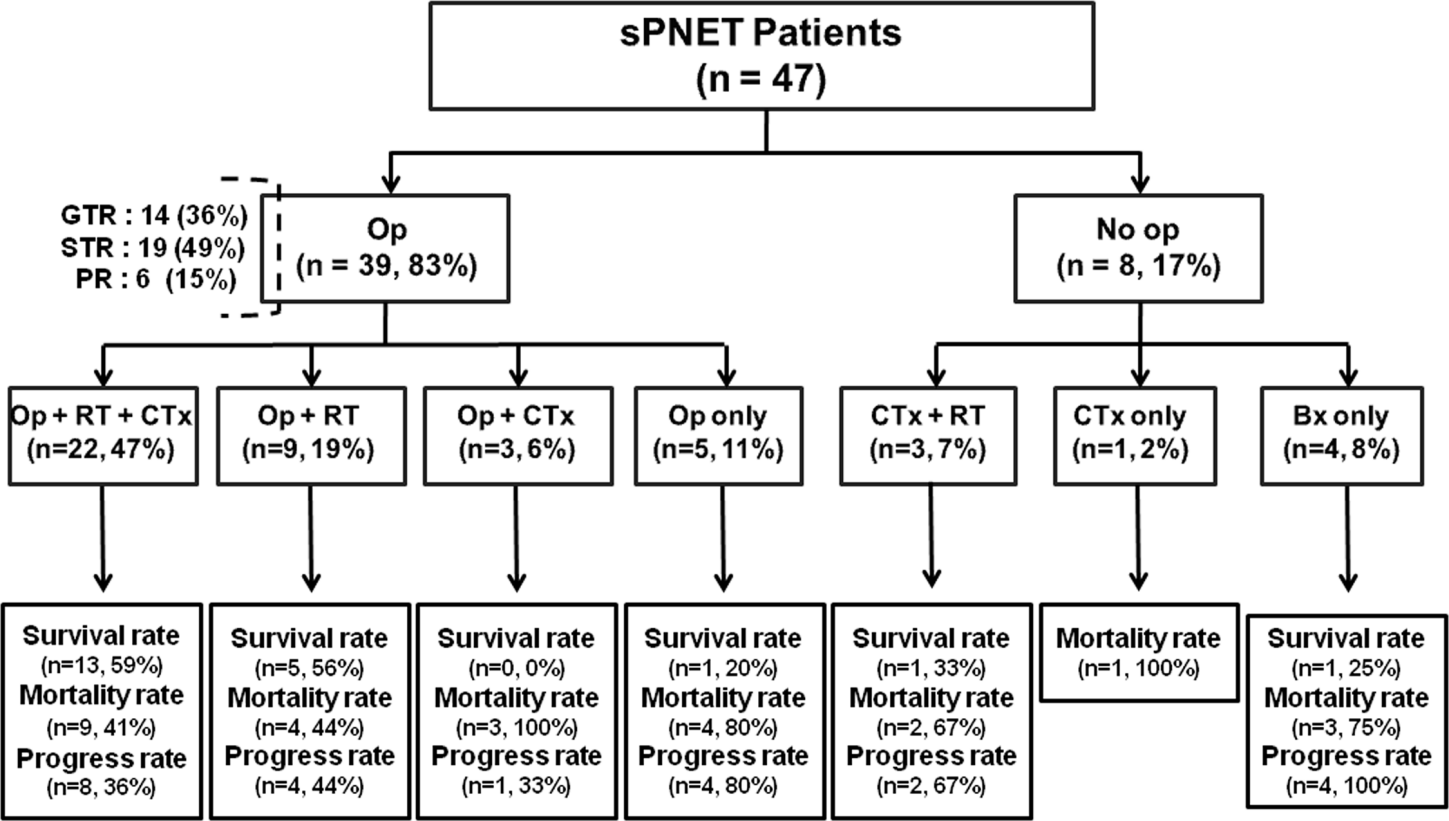
Treatment flowchart for 47 patients with sPNET. Op, operation; RT, radiotherapy; CTx, chemotherapy; Bx, biopsy; GTR, grossly total resection; STR, subtotal resection; PR, partial resection.

At our institution, sPNET patients are generally treated with the combined use of surgery and radiotherapy. Twenty-two patients received surgery, radiotherapy, and chemotherapy; 9 patients received surgery and radiotherapy; 3 patients received surgery and chemotherapy; 5 patients received surgery only; and 8 patients were treated without surgery (i.e., chemotherapy and radiotherapy, chemotherapy alone, or biopsy alone). Among the 39 patients who underwent surgery, GTR was performed in 14 patients (36%), STR in 19 patients (49%), and PR in 6 patients (15%). Enhancement in T1-weighted postoperative magnetic resonance imaging (MRI) images was considered residual tumor. Treatment characteristics are summarized in Fig 1.

Chemotherapy is also given concomitantly depending on the patient’s condition and tumor characteristics. Chemotherapy was performed in 29 out of 47 patients. Diverse chemotherapy regimens were used according to patient age and disease status at the time of diagnosis for a long time period in our study. The most commonly used chemotherapy regimens were Cisplatin, Etoposide, Cyclophosphamide/Ifosfamide, and Vincristine.

### Radiotherapy

Radiotherapy regimens consisted predominantly of craniospinal irradiation (CSI) and the application of external beam radiotherapy to the tumor bed. The use and timing of radiotherapy was determined by a radiation oncologist and largely based on the patient’s functional status, age, and physician preference. The choice between focal radiotherapy with and without CSI was influenced by the age of the patient; those younger than 3 years of age were more likely to receive focal radiotherapy without CSI.

Among the 34 patients who received radiotherapy, 31 were treated with CSI at a median dose of 36 Gy (range, 23.4–39 Gy) and focal radiotherapy at doses ranging from 14–36 Gy. One patient received whole-brain radiotherapy with focal boost radiotherapy (total 45 Gy). The median dose of total radiation was 54 Gy (range, 40.2–60 Gy). Four patients were treated with CSI at a dose of less than 30 Gy. Two young patients received CSI at a dose 23.4 Gy and additional focal radiotherapy for a total dose of 54 Gy and 59.4 Gy, respectively. Another patient received CSI at a dose of 24 Gy and additional focal radiotherapy for a total dose of 40.2 Gy (this patient was initially diagnosed with germinoma and treated accordingly due to insufficient biopsied material, tumor marker information, and similar features in imaging studies). Finally, another patient received CSI at a dose of 23.4 Gy and additional focal radiotherapy for a total dose of 59.4 Gy.

### Immunohistochemistry and assessment of LIN28A, OLIG2, and Rad51

**Fig 2:**
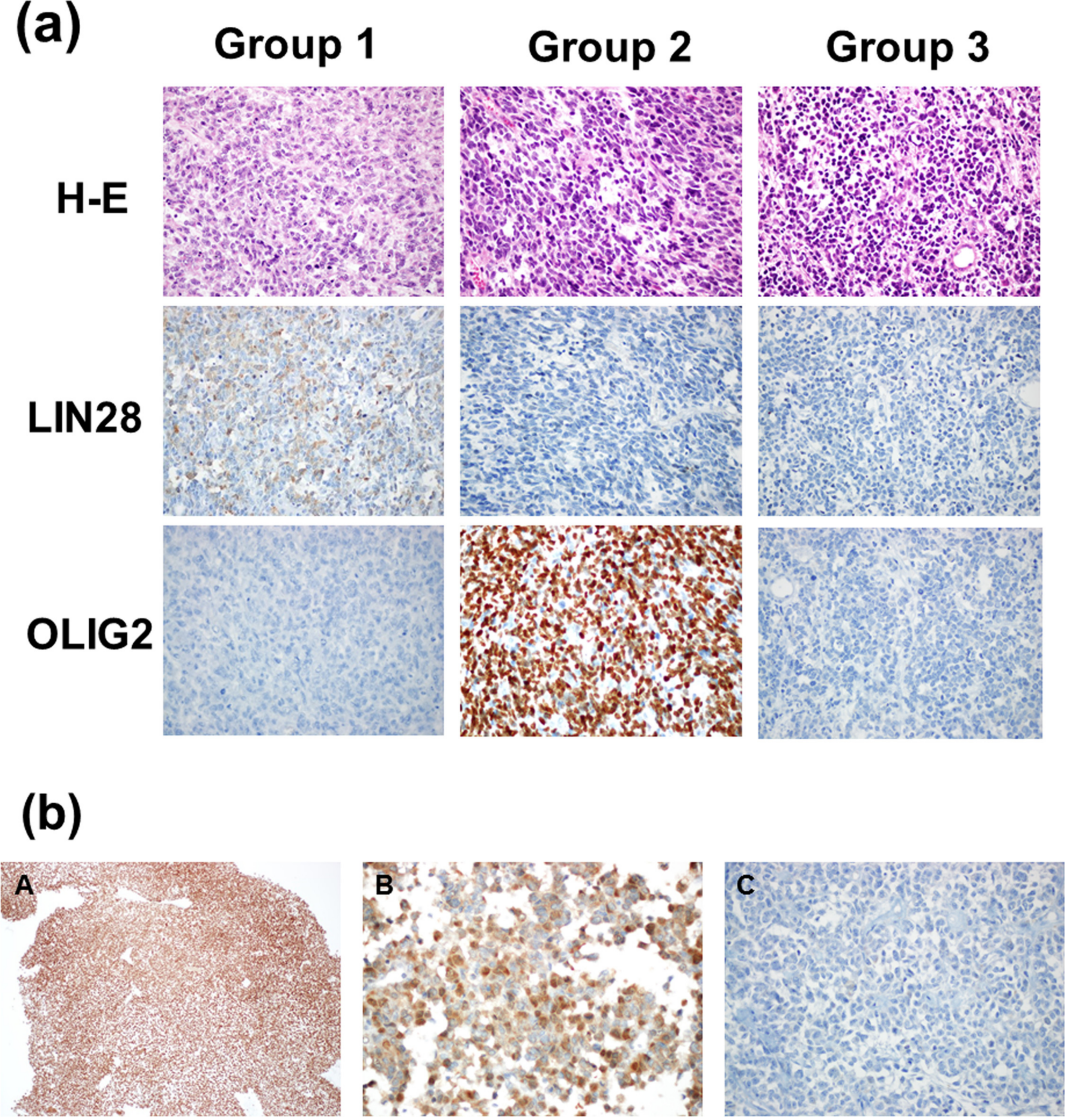
(a) Group 1, 2, and 3 classifications were based on immunohistochemical staining of LIN28 and OLIG2 (magnification: 400×). (b) All tissue specimens showed positive nuclear INI-1 staining (A, magnification: 100×). Tissue specimens were positive (B, magnification: 400×) or negative (C, magnification: 400×) for nuclear RAD51staining.

Of the 47 patients, tissue samples were available for 29 patients. All of these patients showed positive INI-1 staining (clone BAF47, BD Bioscience, San Jose, CA, USA), ruling out the possibility of atypical teratoid/rhabdoid tumor. Archived formalin-fixed, paraffin-embedded tissue slides underwent immunohistochemical staining for LIN28A (clone A177, Cell Signaling, Danvers, MA, USA, 1:100 dilution), OLIG2 (polyclonal, R&D Systems, Minneapolis, MN, USA, 1:200 dilution), and RAD51 (clone H-92, Santa Cruz, Santa Cruz, CA, USA, 1:25 dilution) following protocols for the Ventana Discovery XT automatic platform (Ventana Medical System, Tucson, AZ, USA). Evaluation of cytoplasmic LIN28 and nuclear OLIG2 expression was performed by two pathologists (SH Kim and J Choi) according to the methods of Picard et al.[11]. High LIN28 and low OLIG2 expression was classified as Group 1, high OLIG2 and low LIN28 expression was classified as Group 2, and low or absent LIN28 and OLIG2 expression was classified as Group 3 (Fig 2A).

Evaluation of nuclear RAD51 immunohistochemistry was performed according to the methods of Qiao et al.[17]. We calculated a positive-cell index (PCI) defined as the proportion of positively stained tumor cells. At least 200 tumor cells were counted for each specimen. Using this method, a PCI of 10% was identified as the optical cutoff; samples showing a PCI<10% were considered negative, whereas those showing a PCI>10% were considered positive (Fig 2B).

### Statistical analysis

The Kaplan-Meier method was used to calculate the rates of overall survival (OS) and progression-free survival (PFS). OS was calculated from the date of the initial diagnosis to the date of death due to any cause, or the date of the last follow-up. PFS was calculated from date of the initial diagnosis to the date of recurrence or progression, or the date of the last follow-up date for patients who did not experience these events. Differences in OS and PFS between groups were estimated using log-rank tests and Cox regression. A two-tailed *p*-value<0.05 was considered statistically significant. All statistical analyses were carried out using SPSS version 20.0 (SPSS Inc., Chicago, IL, USA).

## Results

### Survival and prognostic factors

We first analyzed disease progression and survival rates depending on treatment modality (Fig 1). Survival rate was the highest in the surgery, radiotherapy, and chemotherapy group. Eight patients (36%) experienced disease progression, and seven of these patients died (one patient experienced disease progression but lived after salvage treatment). The death of the two patients without disease progression/recurrence was due to unknown causes and subdural hemorrhage, respectively. In the surgery and radiotherapy group, four patients (44%) experienced disease progression and died. In the surgery and chemotherapy group, disease progression was confirmed for only one patient (33%), but all three patients died at 2.6 months, 3.9 months, and 22.7 months, respectively, due to sepsis after chemotherapy. In the surgery only group, four patients (80%) experienced disease progression, and three of these patients died. The non-surviving patient without disease progression had a persistent tumor and died after 33 days of semicoma status after surgery. In the chemotherapy and radiotherapy group, two patients (67%) experienced disease progression and died. The surviving patient with no disease progression was 7 years old when she was diagnosed with PNET of the pineal gland. She received 36 Gy of CSI, 19.8 Gy of additional local boost, and chemotherapy. The one patient in the chemotherapy only group (100%) died of the disease after 6.74 months. In the biopsy only group, all four patients showed disease progression, and three of them died.

**Fig 3:**
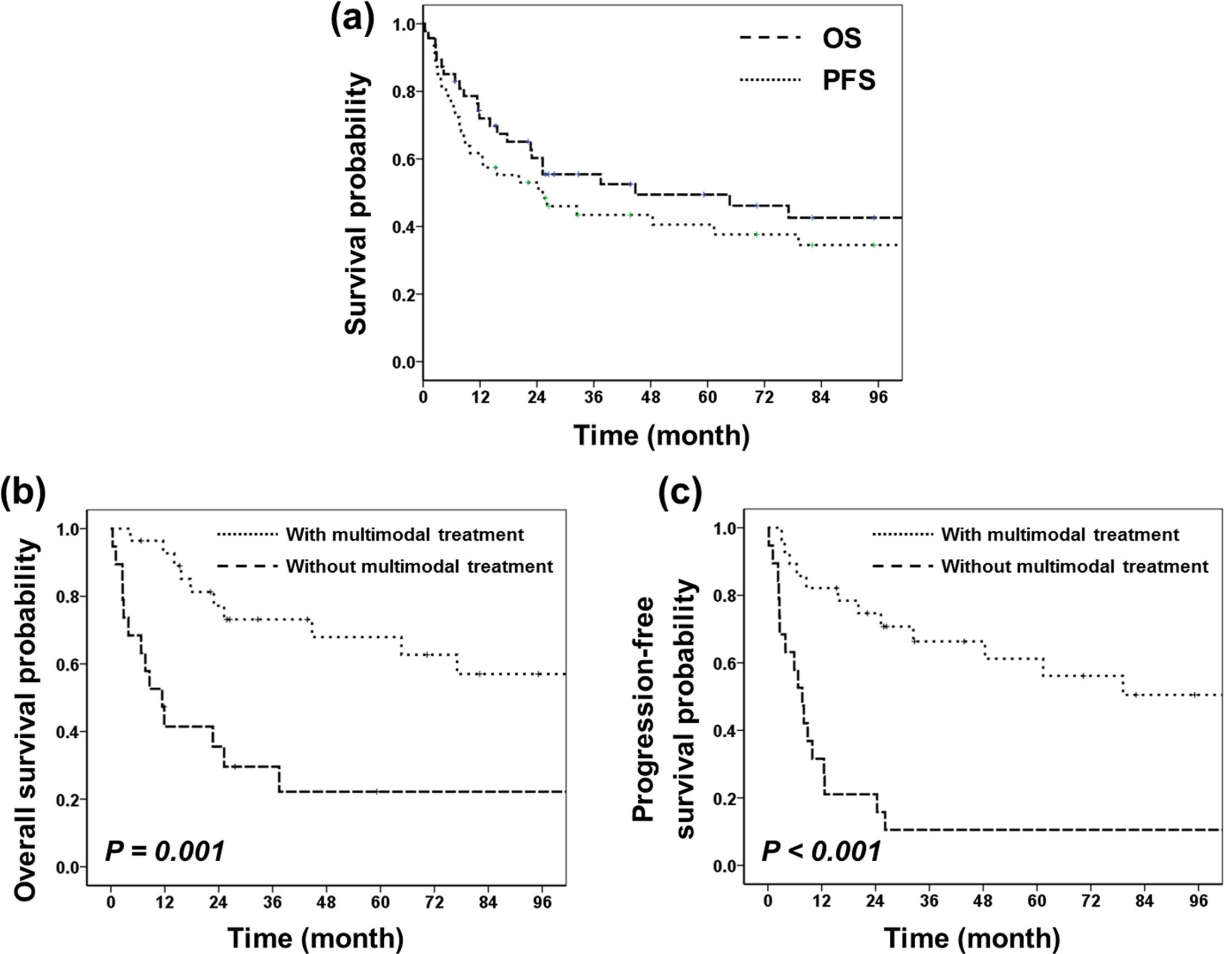
(a) OS and PFS for all patients (n = 47). (b) OS depending on inclusion of multimodal treatment. (c) PFS depending on inclusion of multimodal treatment. Multimodal treatment consisted of radiotherapy and surgery (≥subtotal resection), with or without chemotherapy (with multimodal treatment, n = 24; without multimodal treatment, n = 23).

Considering all patients, the 3- and 5-year OS were 55.5% and 49.4%, and the 3- and 5-year PFS were 43% and 40% (Fig 3A). Patients who initially planned to receive multimodal treatment without interruption (radiotherapy and surgery (≥STR), with or without chemotherapy) showed significantly higher 3- and 5-year OS and PFS than patients without multimodal treatment (OS: 77.1% and 71.2% vs. 33.2% and 27.7%, *p* = 0.001; PFS: 68.8% and 63.1% vs. 17.4% and 17.4%, *p*<0.001, respectively; Fig 3B and 3C). Multimodal treatment was also significantly associated with better 3- and 5-year OS and PFS (OS: 84.1% and 77.1% vs. 40.5% and 33.8%, *p* = 0.006; PFS: 73.9% and 67.2% vs. 21.1% and 21.1%, *p* = 0.001) among patients who were older than 3 years of age.

In univariate analysis, age (≥3 years), surgical extent (≥STR), and radiotherapy were significant prognostic factors for OS (*p* = 0.009, 0.014, and <0.001) and PFS (*p* = 0.03, 0.009, and <0.001). In multivariate analysis, radiotherapy was an independent prognostic factor for OS (*p* = 0.001) and PFS (*p*<0.001) (Table 2).

**Table 2:** Prognostic factors for OS and PFS as shown by univariate and multivariate analyses.

	OS				PFS			
		Univariate	Multivariate			Univariate	Multivariate	
Variable	5-year (%)	*P* value	HR (95% CI)	*P* value	5-year (%)	*P* value	HR (95% CI)	*P* value
**Sex**								
** Male**	54	0.70			41	0.80		
** Female**	47				42			
**Location**								
** Pineal gland**	55	0.80			49	0.80		
** Others**	46				36			
**Age**								
** < 3yr**	14	0.01	0.35 (0.13–0.95)	0.04	14	0.03	0.48 (0.18–1.25)	0.13
** ≥ 3yr**	56		0.09 (0.01–0.60)^*^	0.01^*^	40			
**KPS**								
** < 80**	37	0.09			32	0.06	2.81 (1.04–7.62)^*^	0.04^*^
** ≥ 80**	59				47			
**Multiplicity**								
** Yes**	66	0.90			33	0.70		
** No**	49				41			
**Metastasis**								
** Yes**	52	0.60			42	0.80		
** No**	48				41			
**STR**								
** Yes**	61	0.01	0.58 (0.23–1.46)	0.24	52	0.01	0.78 (0.32–1.90)	0.58
** No**	22		0.13 (0.04–0.43)^*^	0.00^*^	14			
**RT**								
** Yes**	65	<0.001	0.21 (0.08–0.52)	0.00	56	< 0.001	0.14 (0.06–0.37)	< 0.001
** No**	10				0		0.11 (0.04–0.37)^*^	< 0.001^*^
**CTx**								
** Yes**	50	0.60			43	0.20		
** No**	49				35			
**Rad 51**								
** Positive**	15	0.05^*^			0	0.02^*^	5.73 (1.65–19.89)^*^	0.00^*^
** Negative**	63				56			
**LIN28, OLIG2 group**			Group 1+2 vs. 3				Group 1+2 vs. 3	
** Primitive neural (group 1)**	0	0.08^*^	0.19 (0.06–0.65)^*^	0.01^*^	0	0.01^*^	0.17 (0.05–0.52)^*^	0.00^*^
** Oligoneural (group 2)**	43				29			
** Mesenchymial (group 3)**	49				41			

Abbreviations: OS, overall survival; PFS, progression-free survival; KPS, Karnofsky performance status; STR, subtotal resection; RT, radiotherapy; CTx, chemotherapy; HR, hazard ratio; CI, confidence interval

^*^: Results from subgroup analysis including 29 patients with available tumor biopsies.

### Patterns of treatment failure

Disease progression was observed in 23 patients (48.9%), with eight showing persistent disease and 14 experiencing recurrence after a response to treatment. The most common site of disease recurrence was the primary tumor site (93%). Twenty-one patients showed disease progression in the local CNS, whereas only one patient showed disease progression in the distant CNS. Patterns of treatment failure were not significantly related to radiotherapy dose or mode of treatment.

### Patients less than 3 years old

The seven patients who were younger than 3 years old showed much worse prognosis than those who were older than 3 years old. Three patients received multimodal treatment including radical surgery, radiotherapy including CSI, and chemotherapy. In our institution, we attempted radiotherapy after resection and/or chemotherapy once the patient was older than 3 years, as long as the patient was tolerable to radiotherapy (2: biopsy only, 1: resection only, 1: chemotherapy only). The 3-year OS and PFS of the three patients who received multimodal treatment were higher than those of the patients who did not receive multimodal treatment, but these differences were not statistically significant (OS: 84.1% vs. 33.3%, *p* = 0.069; PFS: 73.9% vs. 33.3%; *p* = 0.123).

### Molecular markers

Next, we examined tumor biopsies from patients (n = 29) using immunohistochemistry to investigate the patterns and intensities of LIN28, OLIG2, and Rad51 expression.

#### LIN28 and OLIG2

We classified sPNET patients into three molecular subgroups based on differential expression of tumor cell lineage markers LIN28 and OLIG2 according to the methods of Picard et al.[11] Two patients (7%) belonged to Group 1, 7 patients (24%) belonged to Group 2, and 20 patients (69%) belonged to Group 3. We found no differences in sex, age, or treatment modality between the three molecular subgroups.

**Fig 4:**
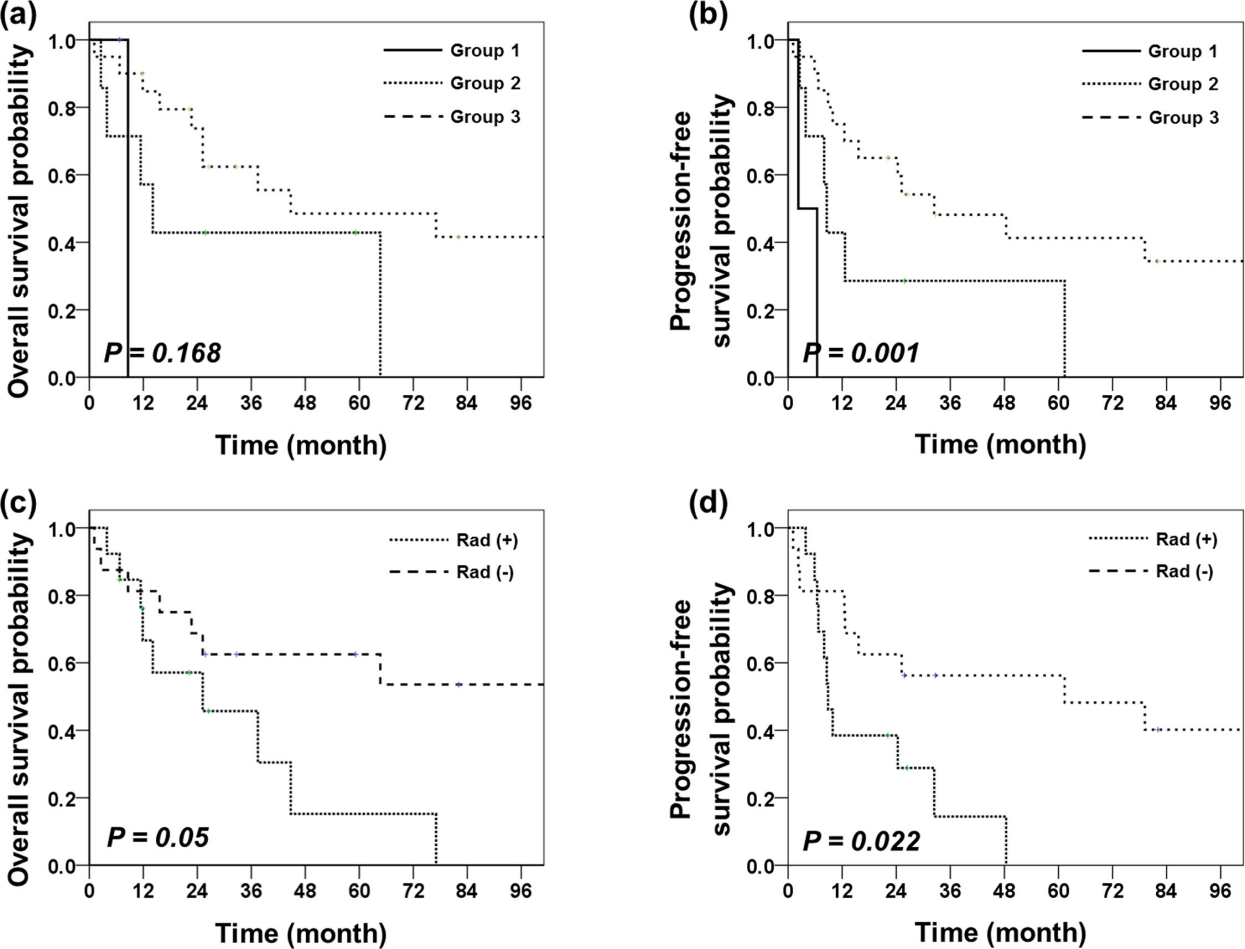
(a) OS depending on LIN28/OLIG2 expression level. (b) PFS depending on LIN28/OLIG2 expression level (Group 1: n = 2, Group 2: n = 7, Group 3: n = 20). (c) OS depending on Rad51 expression level ((+): n = 13, (-): n = 16)). (d) PFS depending on Rad expression level.

Patients in Group 1 had the poorest OS (median survival: 7.66 months for Group 1, 14.4 months for Group 2, and 29.58 months for Group 3) and PFS (median survival: 4.35 months for Group 1, 8.57 months for Group 2, and 25.83 months for Group 3; Fig 4A and 4B). Univariate analysis showed that PFS was significantly poorer for Group 1 + 2 than for Group 3 (*p* = 0.013) and significantly poorer for Group 1 than for Group 2 + 3 (*p* = 0.001). A similar pattern of differences between groups was observed for OS, but these differences were not statistically significant (Group 1+2 vs. 3, *p* = 0.084; Group 1 vs. 2+3, *p* = 0.231). We also found differences between groups in the incidence of tumor metastases at initial diagnosis. Patients in Group 3 had higher incidence of disseminated disease at diagnosis (5/20, 25%) than the other groups (Group 1: 0/2, 0%; Group 2: 1/7, 17%), but it was not statistically significant.

#### Rad51

We found an elevation of Rad51 expression in 45% of tumor specimen (13 patients). No significant differences were found between patients with elevated or low/absent Rad expression with regard to age, surgery extent, or radiotherapy.

Univariate analysis showed that patients with elevated Rad51 levels exhibited significantly worse 5-year OS and PFS than patients with low or absent Rad51 (OS: 15.2% vs. 62.5%, *p* = 0.050; PFS: 0% vs. 56.3%, *p* = 0.022). Disease progression was found in 69% of patients in the Rad (+) group and 38% of patients in the Rad (-) group (Fig 4C and 4D).

When possible, prognostic parameters (i.e., age ≥ 3 years old, surgical extent, use of radiotherapy, expression of molecular markers, and Karnofsky Performance Score (KPS)) were included in multivariate analysis. LIN28/OLIG2 expression, STR, and age were significant prognostic factors for OS, and LIN28/OLIG2 expression, Rad51 expression, use of radiotherapy, and KPS were significant prognostic factors for PFS (Table 2).

## Discussion

We found that multimodal treatment improves outcomes for patients with sPNETs, especially when radiotherapy including CSI and at least subtotal surgical resection were part of the treatment regimen. Because local disease progression was the primary recurrence pattern, local control should be preferred to improve survival, with a sufficient extent of surgery and proper total dose of radiotherapy for each patient. These findings are consistent with many previously published results [3–7] but are in contrast to a recent retrospective analysis that suggests that OS does not correlate with the extent of resection [21]. Other studies show that factors including the age of less than 3 years, metastasis, tumor necrosis, and tumor dissemination are associated with poor prognosis, especially for childhood cases of sPNETs. Similarly, we found that age, surgical extent, and radiotherapy were significant prognostic factors in both univariate and multivariate analyses. In particular, the inclusion of radiotherapy in the treatment regimen was the most powerful prognostic factor. Additionally, we also found that specific molecular markers are significant prognostic factors for sPNETs.

Even with good prognostic factors and aggressive treatments, sPNET patients have a poor prognosis, with previous studies reporting a median survival time ranging from only 8 to 23 months. For children, the reported 3- and 5-year survival rates are 34–57% and 18–38%, respectively [3–7, 10, 22]. There have been promising results utilizing myeloablative chemotherapy and autologous hematopoietic cell rescue (without radiotherapy) in young patients [23–25]. Survival rates for adults, however, are worse than those for children. Gandhi et al. reports a median survival time of 16 months in adults with sPNETs, with 1-, 2-, and 5-year survival rates of 55.3%, 35.0%, and 16.5%, respectively [26]. At our institution, the 3- and 5-year OS were 55.5% and 49.4% considering all patients, which are higher rates than those reported by other studies. Furthermore, we found that multimodal treatment without interruption was associated with particularly high 3- and 5-year OS of 73.1% and 67.9%. This multimodal treatment, which includes maximum surgical resection and radiotherapy, also improved survival even in patients under 3 years of age, which was the group with a very poor prognosis (i.e., 3-year OS and PFS of 33.3% and 33.3%, respectively).

Previous studies have identified several prognostic biomarkers for diverse disease entities, including sPNETs, using immunohistochemical and genetic assays. Rad51 is one of these important molecular markers that has frequently been studied in diverse diseases but not in sPNETs. Rad51 plays a crucial role in the homologous recombination repair pathway by facilitating strand transfer between broken sequences and their undamaged homologues. Its expression is tightly controlled in normal cells, as inappropriate recombination can lead to genomic instability, but its overexpression is observed in the majority of human tumor cells. Several studies demonstrate that Rad51 is not only involved in the progression of carcinogenesis, but also affects resistance to anticancer treatments, particularly by influencing the amount of radiation-induced cell death [27, 28]. Rad51 overexpression correlates with poor prognosis in many cancers [15–18], but its relationship with CNS tumors, including sPNETs, has not yet been thoroughly explored. In one study of glioblastoma patients, Welsh et al. unexpectedly reported that increased Rad51 expression at diagnosis is correlated with improved survival, although the exact pathway is not well understood [19]. Here, for the first time, we analyzed the relationship between elevated Rad51 expression and the survival of sPNET patients. Similar to previous studies of other diseases, we found that elevated Rad51 expression was significantly associated with worse survival rates for sPNET patients, supporting the hypothesis that Rad51 might promote radiation resistance.

The molecular composition of sPNETs is largely unknown. To improve treatment outcomes, the understanding of molecular features and the identification of appropriate therapeutic targets is important. In recent studies, a distinctly aggressive molecular subgroup that showed frequent amplification of chromosome 19q13 miRNA cluster (C19MC) was identified in sPNETs [29, 30]. Picard et al. identified three distinct molecular subgroups of sPNETs using cell-lineage markers LIN28 and OLIG2 [11]. Primitive neural Group 1 tumors, with frequent C19MC amplification and high LIN28 expression, are distinctly aggressive, whereas mesenchymal Group 3 tumors, with low LIN28 and OLIG2 expression, are associated with a high incidence of metastasis. In the present study, we reconfirmed that LIN28 and OLIG2 are promising diagnostic and prognostic molecular markers, even with a small number of patients. New treatment strategies or more aggressive treatments are warranted for patients with Group 1 tumors, as these patients show the worst survival rates even with multimodal treatment. Such molecular markers will help predict prognosis and enable tailored treatments for each subgroup of patients.

Nonetheless, this study has several limitations that should be noted. First, due to the rarity of sPNETs, our sample size was relatively small. Second, this is a retrospective review spanning a long time period, during which major advances in surgical technique, radiation delivery, and chemotherapeutic agents have occurred. However, we selected only those patients who received surgery and radiotherapy at our institution with exact treatment information to minimize bias in this analysis. Third, due to the variability in the definition of PNET pathology, patients with heterogeneous prognosis might have been included in our analysis. Even with these kinds of heterogeneity, however, our study showed overall excellent survival outcomes of multimodal treatment and identified valuable prognostic molecular markers.

As already known, we observed a positive trend toward improved OS with more extensive resection and radiotherapy including CSI. Thus, sPNETs should be treated aggressively after multidisciplinary discussion. The study of molecular markers will help to classify CNS PNETs and to identify high-risk subgroups that need more aggressive treatments tailored to their specific biological profiles. We confirmed the role of LIN28 and OLIG2, which were previously proposed as prognostic molecular markers for sPNETs, and also discovered the role of Rad51 in sPNET prognosis. Prospective studies or larger cohort studies will help unravel the relationship between these molecular markers and clinical outcomes and determine ideal therapeutic strategies. In summary, we examined multiple prognostic molecular markers and their clinical meaning, and our study is the first to identify the role and prognostic importance of Rad51 in sPNETs.
